# Impact of alcohol marketing on drinkers with Alcohol use disorders seeking treatment: a mixed-method study protocol

**DOI:** 10.1186/s12889-020-08543-6

**Published:** 2020-04-07

**Authors:** Morgane Guillou-Landreat, Antoine Dany, Jean Yves Le Reste, Delphine Le Goff, Amine Benyamina, Marie Grall-Bronnec, Karine Gallopel-Morvan

**Affiliations:** 1 Addictive disorders Unit, CHU Brest, Brest, France; 2 EA 7479 SPURBO, University of Brest, Brest, France; 3 HUGOPSY network, Brest, France; 4 Hôpital Paul Brousse, APHP, 94800 Villejuif, France; 5 Addictive Medicine and Psychiatry Department, CHU Nantes, Nantes, France; 6 INSERM UMR 1246, SPHERE, Methods in Patient-Centered Outcomes and Health Research, Nantes and Tours University, Nantes, France; 7 EHESP, School of Public Health, CREM UMR CNRS 6211, Rennes, France

**Keywords:** Alcohol, Alcohol use disorder, Marketing, Advertising, Counter-marketing

## Abstract

**Background:**

The marketing of alcohol influences patterns of alcohol consumption. Existing studies have focused, for the most part, on adolescents and the links between exposure to marketing and alcohol initiation. In France, the Evin law, a French exception, was set up in 1991 with the aim of regulating this exposure to marketing, but since 2009 it has been severely compromised. Alcohol consumption causes severe damage, which may be seenfrom 1 standard unit per day and mostly among adults who are regular users of alcohol. In this at-risk population, studies analysing the impact of marketing are sparse.

The specific objectives include (i) the evaluation of the perception of alcohol marketing by patients with an AUD (ii) gaining understanding of the links between alcohol marketing and patients with AUD behaviours (iii) the development of alcohol demarketing strategy in patients receiving AUD coaching.

**Methods:**

Our main objective isto evaluate the impact of marketing on a population with an AUD. The methodology was in 4 steps: step 1 is a pre-test (*N* = 100) selecting type of alcohol consumed and type of marketing stimuli identified by patients aged 18 + with an AUD. Step 2 is a qualitative study (*N* = 20), with in-depth interview, to understand links between alcohol marketing and patients with AUD behaviours. Step 3 is a quantitative study(*N* = 600) to confirm these links and the impact of alcohol marketing on patients with AUD behaviours. Step 4 is an interventional step, including and testing the impact of demarketing intervention on patients with AUD while using the results of the three first steps (*N* = 120).

**Discussion:**

This study will contribute to a better definition of the impact of alcohol marketing on patients with AUD and will enable identification of the determinants of this impact. These data will inform the development of interventions that take into account demarketingstrategies on patients under AUD management.

**Trial registration:**

The Trial registrationregistration number is NCT03876132, and it was registered on the 15th march 2019.

## Background

Alcohol use is a leading risk factor for a global burden of disease and causes substantial damage to health [1–3]. 4·0% of the global burden of disease is attributable to alcohol [4]. Excessive alcohol consumption induces 3.3 million deaths each year, 5.9% of all deaths [5].

Alcohol Use disorders (AUDs), defined by the DSM 5 [6], are characterised by an impaired control over alcohol consumption and a chronic,escalating pattern of alcohol use despite significant damage concerning global health, alsoto the lives of family members and friends, and to society in general [7]. AUDs are among the most prevalent mental disorders, more particularly in high and upper middle-income countries [8, 9]. They are the leading risk factor for premature mortality and disability among 15–49 year-olds around the world [1]. In Europe, in 2010, the number of people affected by AUD was 23 million [10, 11]. It is one of the most important risk factors for morbidity, along with high blood pressure, tobacco use and excess weight [12]. In France it is the second most important cause of preventable mortality after smoking [13]. 47 million French people (aged 11–75) have already consumed alcohol at some point in their lifetime, 43 million in the last 12 months [14]. 10% consume alcohol daily, and 8% have a severe AUD [14].

AUDs are complex chronic disorders and risk factors are individual, environmental, and associated with alcohol [7]. Factors associated with alcohol include alcohol availability, the social role of alcohol and alcohol marketing. In terms of the public health perspective, it is particularly useful to evaluate and monitor these factors because they are modifiable. To combat alcohol misuse, health actors recommend several measures. The SAFER initiatives of the WHO recommend Strenghteningrestrictions on alcohol availability, Advancingand enforcing drinking countermeasures, Facilitating access to screening, providing brief interventions and treatment, Enforcing bans or comprehensive restrictions on alcohol advertising, sponsorship and promotion and Raising prices on alcohol through excise taxes and pricing policies [8]. Alcohol availability, prices and advertising can be included in the larger concept of alcohol marketing. This is defined as a management process from concept to customers, and it includes the four elements called the 4Psof marketing: (1) identification, selection and development of a Product, (2) determination of its Price, (3) selection of a distribution channel to reach the customer’s Place and (4) development and implementation of a Promotional strategy.

The impact of alcohol marketing in a young population, identified as vulnerable [15], is well described. In this subgroup, exposure to alcohol marketing is associated with an earlier initiation, increases drinking intentions and increases consumption and binge drinking [16–19]. It also leads to a normalisation of alcohol consumption and an underestimation of the risks linked to consumption [20].

Drinkers with an AUD are also a vulnerable group, according to Babor et coll [15].. They are vulnerable to health damage [21, 22]; the relative risk of severe liver disease is very high in adulthood among men between 18 and 20 years of age who consume 3 standard units per day [23]; in fact, 90% of the deaths attributable to alcohol concern people with a daily consumption of 5 standard unitsper day or more [1].

Drinkers with AUD are potentially vulnerable to alcohol marketing, but very little work has been done on the links between exposure to alcohol marketing and alcohol consumption in people with an AUD. In literature, some studies focused onheavy users of alcohol, meaning those who were consuming more than the prescribed limits. They react strongly to alcohol cues, and increased alcohol consumption is associated with increased attentional biases towards alcohol cues, which may increase subjective alcohol craving [24, 25]. The young, heavy users of alcohol sawhigher than average alcohol consumption in alcohol ads, but perceived this consumption to be responsible, unless it was excessive [26].

Experimental studies were also conducted, using functional magnetic resonance imaging, on small groups of adolescents (*n* = 15) responding to DSM IV AUD criteria [27], or students(*N* = 46) regularly consuming alcohol with moderate or heavy drinking [28], or on heavy users of alcohol (*n* = 20). They all exposed participants to alcohol images or advertising or films, and concluded that adolescents and students with AUD criteria or higher alcohol consumption had higher brain responsiveness when exposed to alcohol stimuli [27, 28], and higher psychophysiological responsiveness [28]. De Sousa Fernandes Perna et al. showed that, in heavy users of alcohol, public advertising of alcohol elicits striatal activation in the brain’s reward circuit [29].

Only two studies focused on drinkers with an AUD who were seeking treatment [30, 31], but they were both conducted under experimental conditions. Sobell et al. in 1993 exposed 96 drinkers seeking treatment to television programs which included alcohol advertising. They showed that the more severe the AUD, the less confident the patients felt in their ability to control alcohol craving and their desire to drink after the viewing [30]. Witteman developed a mixed methodology in a population of 80 drinkers with an AUD who were seeking treatment and combined an experimental exposure to experimental alcohol promotional films, and a prospective follow-up over 5 weeks, where drinkers self-reported alcohol marketing exposure. They showed a high psychophysiological responsiveness to alcohol cues, and a higher craving after exposure to alcohol cues, proportional to the severity level of their AUD. The drinkers reported being exposed to five alcohol marketing cues per day [31].

Consequently, there is no large-scale study on the impact of alcohol marketing in the population of drinkers with an AUD. Previous studies focused on a young population or on heavy drinkers [24–26, 32], but only two focused on drinkers with an AUDwho were seeking treatment [30, 31]. They were all conducted on a small number of participants and,in the majority of cases,under experimental conditions [26–28, 30, 31]. They all limited their exposure to a single alcohol marketing tool, either posters or promotional films, which does not reflect the broad scope of marketing [26–28, 30–32]. Furthermore, none of them used a qualitative study to explore the drinkers’ perception of the marketing and to understand the links between marketing and drinkers’ behaviour. Finally, none of these studies wasconducted in France, however the French setting is specific where alcohol marketing is concerned. In 1991, the Evin law imposed alcohol marketing regulations [33], but this law has been debated many times and it has been progressively deconstructed so that, nowadays, there is broad exposure to alcohol marketing [20].

Drawing on thesefindings, this study was designed in collaboration with researchers specialising in addictive disorders, in social marketing and in primary care. It is funded by the French National Cancer Institute (INCA). This study will evaluate the impact of alcohol marketing on drinkers with an AUD who are seeking treatment, using a mixed methodology in four steps. The hypotheses are that 1/ patients with an AUD are sensitive to alcohol marketing strategies because of their AUD and in proportion to the severity of their disorder, 2/ the impact of marketing tools differs according to the type of technique used (ads in public places, product placement in films and series, prices, promotions, etc.), 3/ the marketing of alcohol products influences different factors that contribute to the consumption of drinkers with an AUD who are seeking treatment (affective reactions, social norms, etc.), 4/ exposure to marketing of alcohol brands may be a triggering factor, sustaining or aggravating a higher craving among this particular category of consumers, and that, 5/ the development of interventions that focus on the manipulation of consumers and the effect of the marketing of alcohol products (i.e. demarketing strategies). This could have relevance for drinkers with AUD who are seeking treatment in order to increase their resistance skills and prevent them from relapse.

This article presents the protocol; it follows the Standard Protocol items: recommendations for Interventional Trials (SPIRIT) 2013 guidelines [34].

## Methods/design

### Aim of the study

The primary objective is to evaluate the impact of alcohol marketing on drinkers with an AUD who are seeking treatment.

The secondary objectives are 1/ to determinethe impact of marketing according to type of marketing tools, 2/ to identify how the marketing of alcohol products influences consumption in drinkers with an AUD who are seeking treatment, 3/ to evaluate the links between exposure to marketing and alcohol craving, 4/ to develop interventions that focus on the manipulation of consumers and the effect of the marketing of alcohol products (i.e. demarketing strategies) for drinkers with AUD who are seeking treatment in order to increase their resistance skills and prevent relapse.

### Design and setting

A mixed methodis applied for this study**,** using both qualitative and quantitative research methods to investigate the topic. The combination of a qualitative and a quantitative approach in succession offers the opportunity to answer both the question of exploration and that of confirmation of hypothesis. A Mixed Methods approach is useful when it comes to answering complex research questions, such as the impact of alcohol marketing on drinkers with an AUD who are seeking treatment [35]. Qualitative methods enable investigators to acquire an in-depth understanding of issues, particularly as previous research did not allow us to identify the influence of marketing and which types of marketing tools were those most identified by drinkers. A quantitative approach will make it possible to interview a large sample of drinkers with an AUD,who are seeking treatment, about alcohol marketing (*n* = 600) and also to assess research hypotheses.

### Characteristics of the participants and description of materials

Drinkers aged 18 and over,presenting a moderate or severe AUD, identified by AUDIT score (> 9) and confirmed by the DSM5 criteria (at least 4 criteria of the DSM alcohol use disorder section in the previous year), who are seeking treatment and who have given their written consent will be included. Non-inclusion criteria are:underage drinkers, vulnerable adults and people who do not understand the French language (written or spoken). No care or interventions were prohibited during the study, except a participation to a study on alcohol in the same period.

For step 1 (preliminary study), step 2 (qualitative study), and step 4 (interventional study) drinkers with an AUD who are seeking treatment in Brest University Hospital will be included.

For step 3 (quantitative study), drinkers with an AUD who are seeking treatment in BrestUniversity Hospital, in Nantes University hospital and the Paul Brousse University hospital in Paris will be included.

**Fig. 1:**
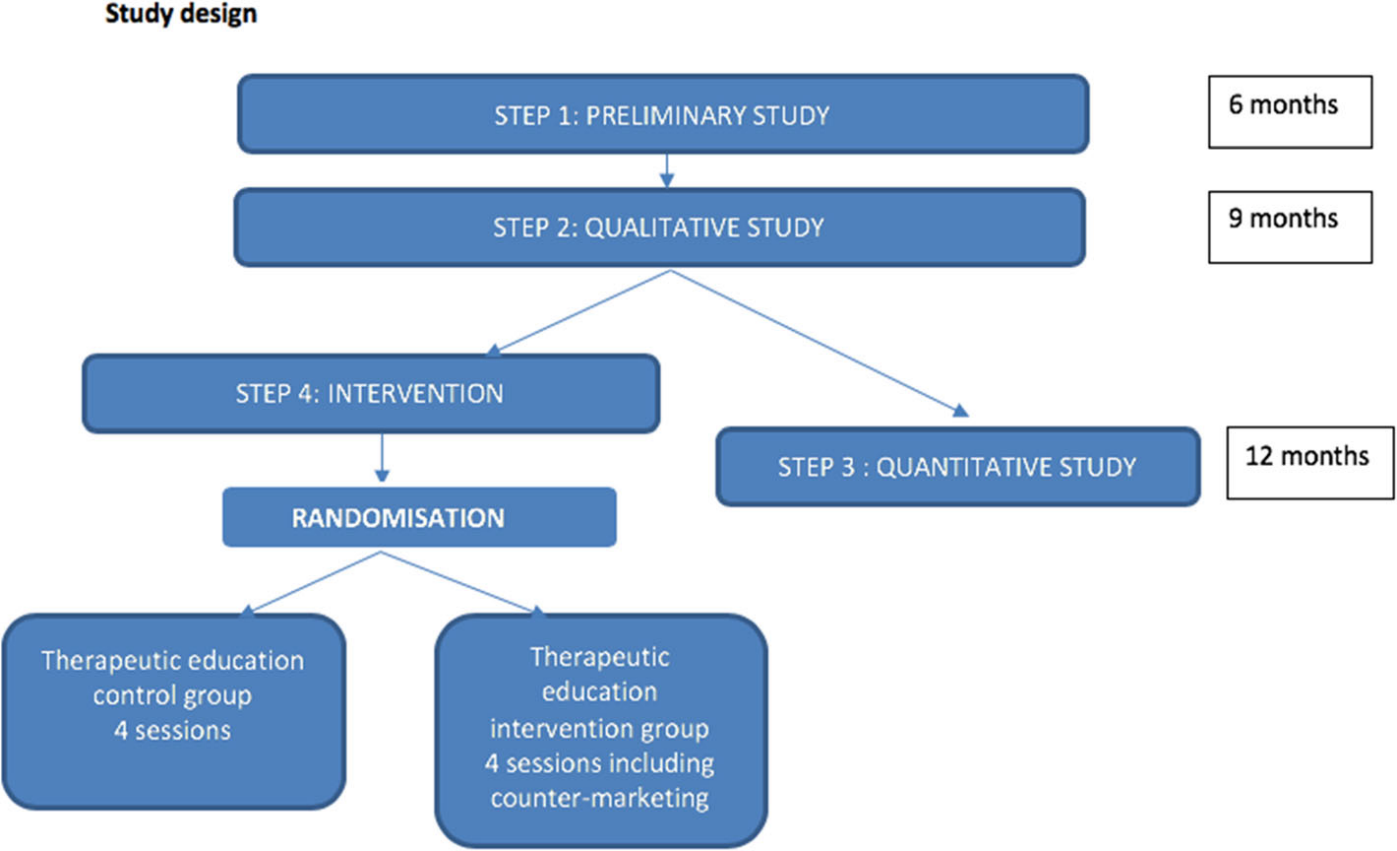
Study design

Study design is represented in Fig. 1**.**

#### STEP 1: preliminary study

A preliminary study is being conducted in the Addiction Centre at Brest University Hospitalamong drinkers with an AUD who are seeking treatment. The objective of this step is to identify the type of alcohol usually consumed by patients and the type of marketing strategy and tools identified by patients.

A questionnaire will be constructed and tested in a pilot survey. The questionnaire will be constructed in collaboration among researchers who are specialists in addictive disorders, social marketing, and the primary care population. The themes of this questionnaire will be: 1/ sociodemographic data 2/ history of AUD: familial and personal history, 3/ history of alcohol consumption: the type of alcohol most consumed within the family at the start of alcohol consumption, at the moment when alcohol consumption became a problem and over the previous year, 4/ marketing: type of marketing tools they remember, preferred brand, perception of the influence of alcohol marketing on their consumption.

One hundred drinkerswith an AUD who are seeking treatment will be included for this first step. At Brest University Hospital, after informed consent has been obtained, the questionnaire will be offered to every drinker with an AUD who is seeking treatment and who matches the inclusion criteria,over a 3-month period, by physicians specialising in addictive disorders.

The results of Step 1 will be analysed and will be used to choose the type of marketing tools, the type of alcohol and the type of brand to select for Step 2 and to build the qualitative interview.

#### STEP 2: qualitative study

The objective of the qualitative study will be to question patients thoroughly about different risk situations with alcohol to discover their feelings about, and their sensitivity and exposure to, different marketing tools and advertising for different alcohol brands.

The qualitative method makes it possible to explore and to understand the origin of the behaviours, including personal motivations and barriers regarding exposure to alcohol marketing tools. The objective of this qualitative interview will be to provide an in-depth view of the responses of individuals. The qualitative method has been chosen at this stage because it provides detailed data essential for the analysis of attitudes, perceptions and reactions of individualsregarding alcohol marketing tools.

Qualitative semi-structured interviews will be conducted face to face, to allow each drinker with an AUD to answer in depth, and to allow a longer time for spoken response. The interview formathas beenselected because it is more intimateand reducesthe risk of information bias when compared with a focus group format. The research group will rely on marketing methods which we are already in use [36].

Moreover, for ethical reasons, as this step exposes drinkers with an AUD who are seeking treatment to alcohol cues, the semi-structured interview allows the researcher to tailor the interview to the individual and to manage any possible increase in individual anxiety and craving, caused by the alcohol cues.

A clinical interview guide will be constructed by a scientific committee made up of physicians specialising in addictive disorders, methodologists, primary care specialists and researchers in social marketing. Marketing cues and type of marketing tools will be selected according to the alcoholic beverages most consumed and the attractiveness of the marketing content and on the results of STEP1.

This guide will bring together 1/ sociodemographic data 2/ data concerning the history of AUD 3/ many selected alcohol marketing tools, representing the broad range of marketing in use (posters, promotional films, the internet, product placement in films, street billboards etc.) according to the results of Step 1. The guide will be presented to the interviewees and their opinions and reactions to these stimuli will be gathered. Alcohol craving will be evaluated by using a visual analogic scale at the beginning,and again at the end, of the interview. If craving is higher at the end, drinkers will be invited to stay until the craving is reduced, and coffee and cakes will be offered to them. Indeed, the alternative of food and beverages are among the coping strategies developed in CBT programswhich aim to prevent alcohol relapse, as defined in Mellentin et al. [37]. At the end of the interview, all participants will be given a document, which will be explained to them, presenting data on the manipulation of consumers and the effect of the marketing of alcohol products on alcohol consumption.

The same trained researcherwill conduct all theseface-to-face interviews. Each interview will be recorded. About 20 drinkers with an AUD who are seeking treatment will be included in this step to fulfil a purposive sampling strategy, according to the inclusion criteria, and to achieve data saturation.

Drinkers with an AUD who are seeking treatment at BrestUniversityHospital, and who fulfil the inclusion criteria, will be invited to take part in this study by the physicians specialising in addictive disorders over the 4-month inclusion period. Physicians will obtain written consent;the researcher will contact the participant to check the inclusion and non-inclusion criteria by phone and confirm the meeting for the interview. Interviews will last at least 20 min to ensure data completeness, and an incentive of 20 euros will be given to each participant.

An independent researcher will transcribe the interviews. A thematic analysis of the contents after verbatimtranscription will be conducted by 2 independent researchers. All interviews will be recorded and transcribed before being analysed by researchers. Thematic content analysis is one of the most common forms of analysis in qualitative research [38]. The Analysis will seek connections between different responses and different prevention messages.

Results of Step 2 will make it possible to identify and select the marketing tools, type of alcohol and type of brands that will be tested in the quantitative phase of this work.

#### STEP 3:quantitative observational study

Through the in-depth thematic analysis of the interviews, a questionnaire will be constructed, in collaboration among researchers specialising in addictive disorders, researchers specialising in social marketing and primary care and methodologists.

The questionnaire will collect data regarding 1/ Sociodemographic elements 2/ AUD severity and damage, history of AUD and history of treatment 3/ respondents’ responses to pre-selected alcohol marketing tools and advertising cues during Step 2. The responsesevoked when confronted with these stimuliwill be collected. They should encompass: perceptions, emotions, craving, desire to consume, perceptions of risks related to alcohol. At the end of the questionnaire, all participants will be given an explanatory document presenting data on the manipulation of consumers and the effect of the marketing of alcohol products on alcohol consumption. The questionnaire will be tested in a pilot study to ensure it is comprehensive and understandable.

This questionnaire will be offered by physicians specialising in addictive disordersto drinkers with an AUD who are seeking treatment in three university centres for addictology in France: CHU BREST, CHU NANTES, HOSPITAL PAUL BROUSSE-VILLEJUIF. During an inclusion period of 6 months, drinkers seeking treatment, who fulfilthe inclusion criteria and who have given their written consent, will complete the questionnaire in the addictive disorders centres participating in the study. Six hundred patients will be included in this step.

All the questionnaireswill be analysed (descriptive and univariate and multivariate comparative analysis) by a researcher specialising in statistics.

#### STEP 4: intervention study

The main objective of this randomized, controlled interventional stepwill be to compare the impact of an intervention which includes counter-marketing (focusing on alcohol marketing strategies and manipulation of the consumer) on the ability of drinkers with AUD who seektreatment to take control of their alcohol craving.

This intervention is a therapeutic innovation, based on inoculation theory [39]. It is possible to effectively protect citizens from harmful alcohol consumption by presenting them with this type of marketing and pointing out the ways in which it influences them. The theoretical foundations of “counter-marketing” are derived from inoculation theory, which postulates that the reinforcement of certain beliefs and attitudes - the feeling of being manipulated by marketing - will allow resistance to advertising cues [40]. The principle is based on the biological analogy of vaccination, as in the case of the “Truth” anti-tobacco prevention campaign in the United States, which has been shown to be effective in reducing adolescent smoking [41]. Inoculation messages operate in such a way that people can be protected from future attempts at persuasion by messages that warn them of the impending threat to their standpoint and refute anticipated, opposing arguments [42, 43]. Key mechanisms involved in the inoculation include an increase in the perception of threat and a greater capacity for counter-arguing against persuasive arguments [44]. Meta analyses have shown that narratives have persuasive effects on changesin attitude, intentions and behaviour [45, 46]. They may lead to persuasion by generating emotional connections with a story involving characters or with offering a high level of engagement [47]. Group interactions whichinclude drinkers with AUD who are seeking treatment, will help to deliver narratives with emotional engagement that can facilitate persuasion and inoculation.

The intervention will be offered during the inclusion period of 1 year to every drinker with an AUD who seeks treatment in follow-up,as an out-patient, at Brest UniversityHospitaland who fulfils the inclusion criteria and is consulting at Brest UniversityHospital. Intervention will be presented as a series of therapeutic educational group meetings; the objective is to involve drinkers, to help them to make their decisions, and to help them to reduce alcohol consumption risks. Therapeutic educational groups respond perfectly to these criteria [48].

Four sessions will be organised to facilitate participation and limit the number lost to follow-up. Group meetings will be organised every 2 weeks; they will last 2 h; a nurse specialising in addictive disorders and a physician will lead the groups.

After their consent, a randomisationprocedure will select 2 groups to be compared. Participants will be randomized 1:1: control group / intervention group. The Data Management Unit (UGD) of the Brest CHRU will be responsible for the randomization (computer-generated random numbers).

##### Control group

An educational group therapy focusing on damage caused by alcohol, strategies to control alcohol consumption and to reduce alcohol consumption risks. This will be offeredin 4 sessions (2 h, twice a month), without focusing on manipulation of consumers or marketing strategies.

##### Intervention group

An educational group therapy focusing on damage caused by alcohol, strategies to control alcohol consumption and to reduce alcohol consumption risks. They will be offered in 4 sessions (2 h, twice a month), with the inclusion of a specific session focusing on manipulation of the consumer, marketing strategies and counter-marketing. The session content will be developed according to the results of the qualitative analysis. It will present explanations concerning alcohol marketing strategies and documents regarding alcohol marketing influences. Then participants will be asked to develop cognitive alternatives to identify and fight against the influence of alcohol marketing strategies.

The impact will be analysed to examine thepatients’ perception of their ability to control their alcohol consumption and to control alcohol craving at the end of the group sessions and 3 months later.

### Study design

Study design is presented in Fig. 1.

### Statistical analysis

#### Observational study (step 3)

We will first perform a descriptive analysis of the sample. Quantitative variables will be summarised by mean and standard deviation if they are normally distributed; otherwise, by median first and third quartiles. Qualitative data will be summarised by counts and proportions. Comparative multivariate analyses will be performed to determine variables associated with several patient profiles. With a 5% alpha risk and 95% power, to be able to detect small effect sizes in a multivariate analysis (F-squared of 0.05) using 20 parameters a sample size of 600 patients will be necessary.

#### Intervention study (step 4)

As a secondary objective, inter-group comparative analyses will be performed before and at the end of the intervention on the principal outcome on a small subsample.

## Discussion

This protocol is original because it applies marketing strategies to evaluate the impact of alcohol marketing on groups of drinkers with an AUD who are seeking treatment. There is no large body of data in literature which explores the influence of several types of marketing tools on drinkers with an AUD and uses a mixed methodology. However, this population of drinkers with an AUD does present a target for alcohol marketing strategies. Indeed drinkers presenting an AUD, represent 8% of the French population, but they represent 50% of alcohol purchases in France [49]. The ultimate objective is to be able to propose individual and collective prevention measures to help reduce the risk from alcohol toa vulnerable population of persons with an AUD. The expected impact of this project is multi-faceted. At a preventive level, this study will help to analyse the influence of alcohol marketing, and the modalities of this influence, on drinkers with an AUD who are seeking treatment. It will be one of the first large studies, using a mixed methodology, that will underline the probable impact of alcohol marketing on vulnerable drinkers with an AUD. These results will help to put pressure on public authorities to put in place preventive measures which could reduce the impact of marketing on patients with an AUD. Measures to be considered are: realistic and effective limits on advertising, in terms of the media used as well as the content, but which also take into consideration pricing and brand strategies.

These results would make it possible to discuss current collective prevention measures in France, including the regulation of alcohol marketing. The Evin law was introduced in 1991 [33], to limit exposure to advertisements for alcohol brands among the youngest members of society, with restrictions concerning certain marketing media (posters on the street, television, etc.) and the content of authorised advertising (informative and objective presentation of products...). However, it was gradually deconstructed, with the posting of advertisements being authorised in 1993; advertising on the Internet in 2009 [50]; and, more recently, (2015) an extension of the authorised media and advertising content, as long as the message referred to the gastronomic and cultural heritage and the geographical origin of the product [51]. The result of these developments is that exposure to advertising and marketing of alcohol brands has been growing in France in a significant way and we note their omnipresence in the daily environment of the citizens: posters in the street, promotions, competitions in shops, in the press, on the internet (official sites and social networks), radio and also in films and series etc. [20, 51]. Until now, public health actors failed to take a stand in favour of limiting these developments. Consumer protection is, however, a public duty and there are many public health issues at stake. Given the high social cost of alcohol use disorders, the results of this work seem particularly promising in reducing the societal financial impact of this disorder. With regard to the impact on public health, recommendations will be made to regulatory authorities to improve the supervision of advertising and, more broadly, alcohol marketing practices.

On atherapeutic level, this study will be innovative, with the development of counter-marketing messagesamong drinkers on an AUD program. This study will help to build the content of the educational intervention, including alcohol counter-marketing strategies. In addition, it will make it possible to identify whether this type of focusis useful in treating patients’craving and their perception of their ability to stop consuming alcohol. If this study shows that this kind of intervention improves outcomes for drinkers with an AUD, it will be necessary to develop strategies of this type for drinkers receiving AUD care. Alcohol use disorders are complex diseases:vulnerability factors are a combination of individual factors (gender, psychiatric disorders, familial history of addictive disorders..); environmental factors (social integration, social entertainment, professional and familial status..) and factors linked to alcohol (type of alcohol, social status, damage ..) Alcohol marketing interferes with the environmental factors, because it induces unconscious, daily exposure on multiple occasions, to alcohol marketing cues. It also interferes in factors linked to alcohol, because it maintains the status of alcohol, and positive social attitudes towards it, and it plays down the risks considerably. So in order to reduce the impact of marketing high-risk products,to these vulnerable patients, it would be invaluable to be able to show individuals how they are being affected by marketing without their knowledge, and to provide them with tools to resist such marketing. The hypothesis is that this intervention on demarketing, combined with a therapeutic educational program which targets strategies to control alcohol consumption, will help to give patients a better perception of control. It would help patients with an AUD to better resist alcohol advertising stimuli [40]. Such work has never been carried out in the field of alcohol use, nor on populations with problematic alcohol consumption.

## Data Availability

No complementary data available.

## Abbreviations

AUD: Alcohol use disorder
CBT: Cognitive Behavioural therapy
